# Assessment of medical resident's attention to the health literacy level of newly admitted patients

**DOI:** 10.3402/jchimp.v3i3-4.23071

**Published:** 2013-12-17

**Authors:** Cecile Karsenty, Michael Landau, Robert Ferguson

**Affiliations:** 1 University of Maryland School of Medicine, Baltimore, MD, USA; 2 Internal Medicine, GBMC Internal Medicine Faculty Practice, Greater Baltimore Medical Center, Baltimore, MD, USA

**Keywords:** health literacy, communication barriers, community medicine, teaching, medical residents

## Abstract

**Objectives:**

The objective of this study was to assess communication at the bedside in the emergency room between residents and their patients in order to identify common communication gaps. We also intended to evaluate whether residents for whom English is a second language (ESL residents) communicate less effectively.

**Methods:**

A scorable checklist was developed in order to assess and identify communication gaps between the residents and their patients. Medical students observed the internal medicine and family medicine residents while they admitted patients to the medical service in the Emergency Room. Before this, medical students were trained for two weeks with a senior internist. The role of the medical student was not revealed; rather they were self-described as observers of the admission process.

**Results:**

Over an 8 week period, 71 observations were made of 27 medicine residents. 71 patient intakes were observed, evaluating 27 residents. In 52.1% of these interactions, the residents used medical acronyms when communicating with the patients. During 66.2% of interactions, technical medical terms or expressions were used during the history taking and in only 27.6% of those cases were the terms explained at least partially. Teach back technique was not observed in any of the interactions evaluated. Data was also analyzed based on whether the doctors were ESL residents or native English speakers. ESL residents tended to use significantly more technical language than the native English speakers, but the native English speakers tended to use more acronyms.

**Conclusions:**

How much patients understand of what their doctor says is called “health literacy.” Resident physicians often overestimate their patients’ health literacy, and this leads to communication gaps which have the potential to result in poorer health outcomes for the patients. The checklist developed for this pilot study assessed how well residents tailor their communication to their patients’ health literacy. Our assessment revealed much room for improvement. This checklist can be used as a tool to teach future residents how to better assess and take into consideration their patients’ health literacy level and as a result communicate with patients more effectively.

Health literacy has been a growing topic of interest in medicine over the past 20 years. Health literacy is a measure of the degree to which individuals are able to process and understand basic health information and make appropriate health decisions (1). Studies have shown that low health literacy (HL) is a prevalent problem in the United States, affecting approximately 40% of adults (2). Studies have shown that low HL often results in poorer outcomes for the patients, more frequent emergency department (ED) visits and hospital readmissions (3). Patients with low HL are rarely forthcoming and tend to ask fewer questions to their provider, this along with the physician's use of complex medical terminology may seriously compromise the efficacy of the interaction. One study looking at the use of un-clarified medical jargon with patients showed that 81% of encounters observed contained at least one un-clarified jargon term and that patient comprehension rates were generally low and inadequate (4). Other studies have shown that the use of un-clarified medical jargon causes there to be a wide communication gap between the patient and the provider. Several studies show that there exists a wide gap in doctor–patient understanding of common physiological terms and diseases (5, 6).

Most studies looking at health literacy have assessed communication in the outpatient setting; very few studies have looked at communication between the patient and the provider during hospitalization. Studies have looked at the HL of adults presenting to an urban ED without taking into account whether or not the patients were later admitted to the hospital (7, 8). Another study tried to elucidate the relationship between patient–provider communication and HL in the hospital setting by giving patients a survey 2 weeks after discharge. This study showed that patients with low HL when compared to those with higher HL skills reported that the physician did not explain the process of care clearly (2).

Although low HL is the cause for wide communication gaps, misunderstandings, and medical complications, HL skills are not taught as an integral part of many medical school curricula or residency trainings. A survey of community-based internal medicine residency programs showed that <50% of the programs provided any kind of formal teaching on HL. In the programs that did include such teaching, 75% of the time HL was taught primarily via didactics and in only 25% of cases was role-playing involved (9).

In this study, we are interested in assessing patient–provider communication in the ED during the transition of care from the ED physician to the admitting resident medicine team in an urban ED. Our goal is to assess how well physicians are able to put into practice HL skills in this pressing environment. We were also interested in assessing whether physicians for whom English is their native language struggle less with this issue than physicians who graduated from foreign medical schools.

## Methodology

First, the existing HL literature was reviewed to compile a checklist of items that would assess how well internal medicine and family medicine interns and residents address their patients’ HL. The HL checklist was then edited to only include items that apply to inpatient admission interviews. The two medical students who were scoring the checklist were introduced to the interns and residents as observers interested in shadowing doctors taking patient histories. The two medical students then observed the same doctor–patient interviews for 2 weeks, scored the checklist independently, and compared their scores. By the end of the 2 weeks, the two medical students were scoring the checklist the same way for every patient and behaving in the same ‘fly on the wall’ manner. Data collection began and each medical student observed as many doctor–patient bedside inpatient admission history-taking interviews as possible. This was a convenience study of 71 doctor–patient interviews in which the medical students followed the first available intern and/or resident to the first available patient willing to let the medical students observe their inpatient-admitting interview, most frequently, in the ED. The medical students said as little as possible and recorded as much as possible.

The HL checklist is attached in Appendix and includes items, such as the patient's HL, the number of technical terms or acronyms used by the physician, whether or not the physician explained these terms, whether or not the physician asked if the patient had any questions, and whether the physician taught HL with any of the strategies mentioned in the literature (e.g. did the doctor use pictures or models to explain? Did the doctor use the teach-back technique in which the doctor asks the patient to repeat back what was just explained).

While most of the data collection consisted of recording whether or not events happened, there was a little bit of subjectivity involved in assessing the patient's HL. The patient's HL was measured using a Gestalt summation of the patient's profession, education, how many doctors they see, how well they know their medications, and what technical terms they use unprovoked by the doctor.

The medical students kept the HL checklist and their true motivations hidden from the interns and residents throughout the duration of the data collection. After the data were collected, it was analyzed.

## Results

A total of 71 admission interviews were observed and scored over a 2-month period. Twenty-seven internal medicine and family medicine interns and residents were observed. Eleven of these residents were classified as having English as a second language (ESL) and 16 were native English speakers (non-ESL).

The average patient age for these admissions was found to be approximately 61 and a little over half of the patients admitted were female (Table 1). The median HL was found to be a 3 on a scale of 1–5. A HL level of 3 signifies that the patient had a good understanding of their health conditions if spoken to in plain language and if complex medical terms and procedures were adequately explained.

**Table 1:** Patient sample gender, race, and age distribution

Patient information	
African American	66.2%
Caucasian	29.6%
Other	4.2%
Female	59%
Male	41%
Median patient age	59
Average patient age	61.69

Because of the nature of the HL checklist, the quantitative results focus on the use of medical terminology and abbreviations/acronyms during the admission process. Two analyses were conducted to assess the results. First, the use of medical jargon and acronyms was examined per hospital admission. The use of such terms was then analyzed based on the admitting residents’ ESL or non-ESL status. When looking at hospital admissions, it was found that in over two-thirds of the admission interview, medical terminology was used and was explained at least partially only 27% of the time. Similarly in almost 80% of observed admissions, either medical jargon or acronyms/abbreviations were used during the patient interview. In almost 40% of the observed admissions, both medical terms and acronyms were used by the residents (Table 2). Some examples of medical jargon that were used include: *gram stain, ablation, cardioversion, meningioma, recycle bp, creatinine clearance, dyspnea, syncope, diaphoresis, trend cardiac enzymes, angina, edema, deep vein thrombosis, and echocardiogram*. When interviewing the patients, the residents used acronyms and abbreviations such as: *DVT, ID, GI, echo, cath, ED, PCP, A. fib, A1C, BUN, COPD, AC clinic, UTI, C-PAP, ABG, OB Doc, ICU, DNR/DNI, IRI*.

**Table 2:** Use of medical jargon, technical terms, acronyms, and abbreviations per hospital admission

Variables	No. of interactions	Percentage
Use of technical terms	47/71	66.20
Explanation of tech terms	13/47	27.6
Use of acronyms	40/71	52.1
Admissions where technical language and/or acronyms are used	56/71	78.8
Admissions where technical language and acronyms are used	30/71	39.4

When the use of such language was examined based on the residents’ fluency in English, significant differences were found between the ESL and the non-ESL group. The ESL residents not only tended to use medical jargon more often but also used more medical terms per admissions than the non-ESL residents. By using *t*-tests, it was revealed that there was a statistically significant difference between the use of medical jargon and the amount of medical jargon per interaction when comparing the ESL and the non-ESL groups (Table 3). However, ESL residents tended to use acronyms and abbreviations less often than the non-ESL residents; this difference was not found to be statistically significant at the 5% confidence level.

**Table 3:** Use of medical jargon, technical terms, acronyms, and abbreviations based on admitting resident's English fluency

Variables	ESL	Non-ESL
Interactions with use of medical jargon (p=0.0413) (%)	68.2	50
Explanation of medical jargon (%)	21	29
Average number of medical terms used (p=0.0411)	1.43	0.917
Interactions with use of acronyms/abbreviations (%)	39	45.8
Average number of acronyms/abbreviations used	0.561	0.625

Other key points from the HL checklist yielded interesting results. For example, teach-back was not used in any of the 71 admissions; only 36% of the time did the residents ask the patients if they had any questions and pictures or models to explain medical concepts were used only 5% of the time.

## Discussion

Previous literature has provided evidence that the lack of HL training is a barrier to effective, patient–provider communication, and in turn health outcomes in the outpatient setting. This study tried to assess resident–patient communication at the patient's bedside in the inpatient-admitting interview without a formalized HL training program for residents. Our results suggest that residents do not use the HL techniques proposed by the literature, and that some communication problems may be pervasive. For example, residents, especially ESL residents, use medical jargon in the majority of their patient interviews and most do not explain these terms.

Furthermore, in the majority of interviews, residents did not ask if the patient had any questions. The authors hope that the data presented by this study are used to raise awareness about how doctors can constantly be mindful to ask patients how much they understand to optimize their health care.

Finally, the authors feel that a formalized HL program would improve doctor–patient communication and in turn, health outcomes. The checklist proposed by this study should be used to develop a standardized way to teach HL awareness to residents talking to patients by the bedside. Ideally, after implementing such a training program, studies would provide evidence that the HL training improves patient–provider communication and in turn, patient outcomes.

## Appendix

Is the main interviewer ESL?

How many people are part of the team walking into the patient's room?

How many people participate in the history taking interview?

Is the physical examination explained?

How many people perform the physical examination?

Does the doctor's accent trigger a misunderstanding?

Does the doctor use appropriate grammar/pronunciation/words?

Does the doctor appear to understand the patient?

Does the patient seem to understand the doctor?

If the patient does not seem to understand, does the Dr. realize when patient is not understanding and correct him/herself?

Is there any confusion or a miscommunication between doctor and patient?

Does the doctor clarify after each miscommunication until the question and answer are clear?

Does the doctor prompt the patient to ask questions/clarifications?

Does the doctor use teach back technique?

Does the doctor use models/pictures to explain?

Does the doctor use acronyms?

If the doctor does use acronyms, does he/she explain the terms understandably?

Does the doctor use technical terms, complicated language?

If the doctor does use technical terms, does he/she explain the terms understandably?

Does the doctor speak slowly, loudly, simply and clearly?

Does the doctor listen effectively, show interest in the patient?

Does the doctor allow the patient to finish speaking?

Does the doctor act professionally?

Does the doctor effectively question the family member?

Do the doctor's judgments interfere with patient communication?

Does the patient have a doctor in the hospital?

Does the doctor negotiate an agenda?
